# A study protocol for a randomised trial of adjunct computerised memory specificity training (c-MeST) for major depression in youth: targeting cognitive mechanisms to enhance usual care outcomes in mental health settings

**DOI:** 10.1186/s13063-019-4036-6

**Published:** 2020-01-14

**Authors:** D. J. Hallford, A. M. Carmichael, D. W. Austin, K. Takano, F. Raes, M. Fuller-Tyszkiewicz

**Affiliations:** 1 School of Psychology, Deakin University, 1 Gheringhap Street, Geelong, Melbourne, Victoria 3220 Australia; 2 School of Psychology, Deakin University, 221 Burwood Hwy, Burwood, Melbourne, Victoria 3125 Australia; 3 Division of Clinical Psychology and Psychotherapy, Department of Psychology, Ludwig-Maximilians-University Munich, Leopoldstr. 131, 80802 Munich, Germany; 4 Faculty of Psychology and Educational Sciences, KU Leuven, Tiensestraat 102, Box 3712, 3000 Leuven, Belgium

**Keywords:** Autobiographical memory, Depression, Memory specificity, Memory specificity training, Overgeneral memory, MeST, Depression, Online intervention

## Abstract

**Background:**

Youth depression is highly prevalent and is related to impairments in academic, social and behavioural functioning. Evidence-based treatments are available, but many young people do not respond or sufficiently recover with first-line options, and a significant proportion experience relapse. Consequently, there is clear scope to enhance intervention in this critical period of early-onset depression. Memory specificity training (MeST) is a low-intensity intervention for depression that targets reduced specificity when recalling memories of the past, a common cognitive vulnerability in depression. This randomised controlled trial will assess the efficacy of adding a computerised version of MeST (c-MeST) to usual care for youth depression.

**Methods/design:**

Young people aged 15–25 years with a major depressive episode (MDE) will be recruited and randomised to have immediate access to the seven session online c-MeST program in addition to usual care, or to usual care and wait-list for c-MeST. The primary outcomes will be diagnostic status of an MDE and self-reported depressive symptoms assessed at baseline, 1-, 3- and 6-month intervals. Autobiographical memory specificity and other variables thought to contribute to the maintenance of reduced memory specificity and depression will be assessed as mediators of change.

**Discussion:**

Online provision of c-MeST provides a simple, low-intensity option for targeting a cognitive vulnerability that predicts the persistence of depressive symptoms. If found to be efficacious as an adjunct to usual care for depressed youth, it could be suitable for broader roll-out, as c-MeST is highly accessible and implementation requires only minimal resources due to the online and automated nature of intervention.

**Trial registration:**

Australian New Zealand Clinical Trials Registry, ACTRN12619000234112p. Registered on the 18 February 2019. All items from the WHO Trial Registration Data Set can be found within the protocol.

**Protocol version:**

1.0

## Background

Depression is a highly prevalent mental health disorder affecting 8–20% of all youth [1]. A further 20 to 50% of adolescents report sub-syndromal, yet clinically significant, levels of depression [2]. Youth depression is related to impairment in several life domains, including academic, social and behavioural functioning [3]. Early-onset depression is associated with more chronicity, higher morbidity, higher rates of recurrence and poorer outcomes compared to other age groups [4–7]. Likewise, subclinical levels of depression in youth predict more chronic and severe depression over time [8, 9]. Effective intervention is therefore particularly important during this vulnerable period. Although evidence-based treatments are available, approximately 50% do not remit with first-line options [10], and a significant proportion relapse [3]. Given this, there is clear scope to enhance intervention in this critical period of early-onset depression. This may be achieved through targeting cognitive mechanisms that predict the course of illness.

An impaired ability to retrieve specific memories of personally experienced events is a reliable cognitive marker in depression [11]. Depressed adolescents and young adults show impairments recalling contextually rich, specific autobiographical memories (both positive and negative) that occurred within the space of a day. Instead, young people with depression are more likely to retrieve memories of events extending over longer periods of time, categories of repeated events or abstractions of various experiences [12, 13]. This impairment, termed overgeneral memory (OGM) or reduced autobiographical memory specificity (rAMS), is a robust factor in the onset and maintenance of depression [14]. Deficits in memory specificity have been shown to predict response to treatment [15, 16], and the ability to successfully retrieve specific autobiographical accounts of the past has a vital role in healthy psychological functioning in youth [17]. Importantly, OGM is a vulnerability factor that can influence the longer-term course of depression if not addressed [14, 18, 19].

One evidence-based intervention to remediate this cognitive deficit is memory specificity training (MeST) [20]. MeST involves sustained practice in producing specific autobiographical memories in response to emotional cue words. Feedback is given on whether these responses are specific or not, and participants are prompted to recall detailed and elaborated memories. MeST has been shown to lead to significant improvements in the retrieval of specific memories that is sustained over time [20–23]. Crucially, it also produces significant reductions in depressive symptoms in clinical samples [21–24]. Recently, a computerised format of MeST (c-MeST) has been developed, which provides an easily accessible, low cost option for improving memory specificity [25, 26]. Participants can access c-MeST online, which facilitates repeated practice of retrieving memories over a series of brief modules. Instantaneous, automated feedback is generated to indicate if autobiographical accounts are specific and detailed through a computerised classification algorithm trained using machine learning [25, 27, 28], meaning few resources are needed to implement c-MeST.

Without addressing low memory specificity or OGM, individuals are less likely to experience reductions in depressive symptoms during treatment [15]. Indeed, OGM is predictive of the course of depression, independent of severity [14]. Encouragingly though, memory specificity is demonstrably amenable to change with MeST. Previous research has indicated that youth find autobiographical memory-based interventions to be acceptable and effective [29]. The treatment is low-intensity for youth in the sense that it is not designed to be emotionally arousing, confronting or onerous. Given this, online c-MeST is an ideal candidate for an adjunct intervention that can be delivered alongside other treatments to specifically target this cognitive vulnerability. Enhancing the ability to retrieve specific memories may help to mitigate against maladaptive processes such as cognitive avoidance and rumination [30] and facilitate retrieval of previous experiences to aid in problem-solving [31]. Further, the ability to reappraise beliefs or experiences appears useful for reducing depressogenic thinking [32], and an improved ability to retrieve specific information from autobiographical memory may provide useful content to aid in this cognitive reappraisal. This study will examine the effect of c-MeST in addition to usual care (such as psychological and/or counselling support or medication prescribed for depression) and test whether it enhances treatment effects and reduces risk of depression at follow-up.

The aim of this study will be to conduct the first randomised controlled trial of c-MeST as an add-on intervention to usual care for young adults experiencing a major depressive episode (MDE). This design will allow us to assess whether adding c-MeST as an adjunct intervention to improve memory specificity enhances outcomes on depressive symptoms. We hypothesise that youth that engage in c-MeST, in addition to usual care, will report significantly lower rates of MDE and lower severity of depressive symptoms at follow-up time-points relative to a control group receiving only usual care. It is also hypothesised that the c-MeST group will report significantly greater changes in memory specificity at the follow-up time-points, and that changes in autobiographical memory specificity will predict changes in depressive symptoms.

## Methods/design

### Study design

**Fig. 1:**
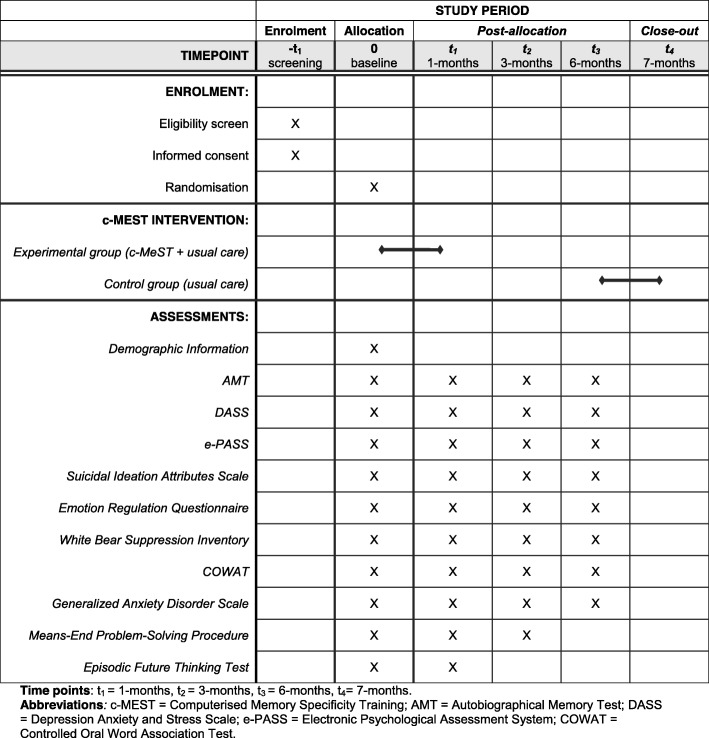
Schedule of enrolment, intervention and assessment

We will conduct an online, randomized controlled trial using a two (condition: usual care and c-MeST, usual care only control group) × four (time-point: baseline, 1, 3 and 6 months post-intervention) factorial design. The usual care control group will complete questionnaires and be offered access to c-MeST at the end of the study. c-MeST has demonstrated efficacy in improving specificity in an online format and therefore this trial will determine whether c-MeST as an adjunct to treatment as usual is superior to treatment as usual only. See Fig. 1 for schedule of enrolment, intervention and assessment. A Standard Protocol Items: Recommendations for Interventional Trials (SPIRIT) 2013 checklist is provided as Additional file 1.

### Participants

The inclusion criteria will be: (i) 15–25 years, (ii) recent or current engagement with a mental health service provider (iii) residing in Australia, (iv) a current diagnosis of an MDE and (v) internet access. The exclusion criteria will be: (i) non-fluency in English and (ii) psychotic, neurodevelopmental and substance use disorders.

Regarding risk, if a participant scores ≥ 21 on the Suicidal Ideation Attributes Scale (the cutoff for risk of suicidal behaviour) then they will receive information regarding helplines/support they might wish to contact. Otherwise, duty of care for risk will be managed by usual risk management protocols through the service they are receiving to increase the generalisability of the findings, comorbid mental health disorders will not be an exclusion criterion of those listed above.

Usual care for participants will vary. As we are recruiting participants at the time of engagement with a mental health service they will be receiving evidence-based treatment for their depressive symptoms (such as anti-depressant medication or psychological/counselling support). Participants will be free to cease involvement in the study at any time.

### Online c-MeST intervention

The c-MeST program will comprise seven sessions based on content from validated methods of improving autobiographical memory specificity in face-to-face [20, 21] and online formats [27]. As previously described in a protocol paper from our group for a phase II trial of c-MeST [28], in each session participants will be provided with a series of positive, negative, and neutral cue words and will be asked to provide a memory of an autobiographical event prompted by the word that is specific in nature. Cue words that have been balanced for frequency, emotional valence and arousal will be presented in a randomised order across intervention sessions. Each cue word will be accompanied by a photo containing people, objects or places thematically similar to the word (e.g., a photo of a person smiling for the cue word *happy*). Participants will also be asked to provide one memory of an event that occurred on that day. Participants will be provided with automated feedback as to whether the response is specific or not via the application of an algorithm developed to classify written autobiographical memories [33]. If the answer is specific, they will be prompted to add spatial-temporal, sensory-perceptual and other contextual details to their responses. When non-specific responses are detected, participants will be asked to re-attempt providing a specific memory. After their first attempt, participants will then be given another two opportunities to provide a specific response before the next cue word is displayed. Although previous studies have shown that brief, time-limited periods of access to c-MeST cause increases in memory specificity [26], feedback from our prior work in c-MeST in depression indicates that some participants required more time, or wanted access over longer periods [28]. Therefore, aside from being advised to avoid completing multiple sessions in one day, completion of modules will be self-paced.

### Primary outcomes

The primary outcomes of the study will be the proportion of participants with a diagnostic status of MDE using the electronic Psychological Assessment Scale (e-PASS) [34] and change in mean self-report depressive symptoms using the shortened version of the depressive subscale from the Depression, Anxiety, and Stress Scale (DASS) [35]. Autobiographical memory specificity will be assessed at all time-points using the standard ten-item Autobiographical Memory Test (AMT), with five positive and five negative cue words [36]. We are primarily interested in the changes in outcome measures between baseline and 3 months.

### Secondary outcomes

Secondary outcomes will all be completed online and include the assessment of suicidal ideation using the Suicidal Ideation Attributes Scale [37], cognitive reappraisal using the Emotion Regulation Questionnaire [38], cognitive avoidance using the White Bear Suppression Inventory [39], verbal fluency using the Controlled Oral Word Association Test (COWAT) [40], general anxiety symptoms using the Generalized Anxiety Disorders Scale [41], problem-solving ability using the Means-End Problem-Solving Procedure [42] and episodic future thinking using the Episodic Future Thinking Test (EFT-T) [43].

### Power calculation

Using G*Power V.3.1 [44], it was calculated that a total sample of 128 participants will be needed. This will be sufficient to detect a moderate-sized, clinically meaningful between-groups effect of *d* = 0.50, with 0.80 power and alpha level of 0.05 (two-tailed), while controlling for amount of usual care treatment (type and amount monitored at each time-point). We will aim to recruit 154 participants at baseline to allow for 20% attrition.

### Statistical analysis

All assessments will be completed using an online format, with data stored online on a secure server accessible by the researchers only. Data entry is completed by participants online and responses are restricted to a predetermined range. The data analyst will be blinded to the condition of participants. Analyses will be on an intention-to-treat basis (i.e., include all randomised participants that complete two or more time-points in the linear mixed models analysis, regardless of the dose of c-MeST or usual care received), with supplementary per protocol analyses (i.e., analysis of participants that have completed all time-points). Linear mixed models will be conducted to assess differences between conditions on the primary and secondary outcomes at each time-point relative to baseline. This statistical method will facilitate inclusion of participants with missing data using a full-information ML estimator. Condition will be modelled as a fixed effect. Time will be modelled as a random effect, grouped within participants. If the groups differ in how much treatment they received in usual care during their participation in the trial, this will be used as a covariate in analyses.

To test mediation effects, primarily whether the effects of group on depressive symptoms over time is mediated through changes in AMT scores over time, analyses will be conducted using a bias-corrected bootstrap test. Power calculations indicate that the sample size will provide power of 0.80 to detect significant indirect effects given small-to-moderate effect sizes between group and depression change, and small-to-moderate effect sizes between changes in AMT scores and depressive symptoms [45]. Given the small number of sites anticipated to be used for recruitment into the present study, site will be dummy coded and included as a covariate in all analyses to adjust for potential between-site differences in outcomes.

### Procedure

Eligible help-seeking youth reporting depressive symptoms will be identified by trained mental health workers through normal intake procedures at participating services. Exclusion criteria of psychotic, neurodevelopmental and substance use disorders will also be assessed at this point. The youth will be informed of the study and asked for permission to be followed up by a member of the research team and invited to participate. They will then be sent an online link with a plain language statement to read (Additional file 2). Consent to participate will be given by clicking an arrow to take them to the next page at the end of the plain language statement to advise of this. They will then complete screening questions to verify eligibility. If participants are eligible, they will be randomised to one of the two conditions (c-MeST and usual care *or* wait-list c-MeST (to be offered in 6 months) and usual care) using computer-generated simple randomisation following completion of the baseline questionnaire. The researchers will be blinded to this process. Participants randomised to c-MeST and usual care will have immediate access to the online c-MeST program and complete online questionnaires at baseline, 1-, 3-, and 6-month time-points. All participation will be online and either involve accessing the memory intervention application or accessing surveys which will be completed via Qualtrics. Participants will receive automated prompts to complete survey measures at each time point. During the first month of access to the intervention, participants will be prompted with automated reminders every second day to engage in the modules. Participants randomised to wait-list c-MeST and usual care condition will complete online questionnaires and receive access to the c-MeST program after the 6-month follow-up. Therefore, all participants will have received usual care and c-MeST by the cessation of the study. No adverse or serious harm is anticipated as part of the online intervention; however, participants will be able to access support through their usual care provided and a list of contact details for support services will also be provided to participants.

## Discussion

Evidence-based treatments for youth depression are available, but many young people do not sufficiently recover with first line options, or relapse. Indeed, this group is among the most susceptible to poorer long-term prognoses if remission is not achieved. Given this, there is clear scope to enhance intervention in this critical period of early-onset depression. Adjunct treatments to usual care provide an opportunity to further improve upon the short- and long-term outcomes for youth. Reduced autobiographical memory is a cognitive vulnerability for depression that can be improved through intervention, and c-MeST offers an online, cost-effective, brief option that has shown promising results in improving the ability to retrieve specific memories.

This study will evaluate the efficacy of c-MeST in addition to usual care in young people with MDE. The current study will provide evidence as to whether engaging in repeated practice of retrieving specific autobiographical memories enhances the effects of usual care on depressive symptoms. The study will also provide insight into whether c-MeST will mediate difficulties related to retrieving specific autobiographical memories (including rumination, cognitive avoidance and executive functioning). If c-MeST is found to be an efficacious adjunct to usual care, the findings will help to inform future studies, such as evaluating the efficacy of c-MeST in other clinical populations vulnerable to OGM.

### Trial status

Recruitment for the trial began on 12 August 2019 with expected conclusion in late 2020.

## Supplementary information

**Additional file 1.** SPIRIT 2013 Checklist: Recommended items to address in a clinical trial protocol and related documents

**Additional file 2.** Plain language statement and consent form

## Data Availability

There are no applicable data since this is a protocol paper.

## Abbreviations

AMT: Autobiographical Memory Test
C-MeST: Computerised memory specificity training
DASS: Depression, Anxiety, and Stress Scale
EFT-T: Episodic Future Thinking Test
E-PASS: Electronic Psychologic Assessment Scale
MDE: Major depressive episode
MeST: Memory specificity training
OGM: Overgeneralised autobiographical memory
RAMS: Reduced autobiographical memory specificity
