# Supplementary material for: A study protocol for a randomised trial of adjunct computerised memory specificity training (c-MeST) for major depression in youth: targeting cognitive mechanisms to enhance usual care outcomes in mental health settings
Source: Trials. 2020 Jan 14;21:85. doi: 10.1186/s13063-019-4036-6 (PMC6961400; doi:10.1186/s13063-019-4036-6)
Supplement: Supplementary file 2 — Additional file 2. Plain language statement and consent form [file 13063_2019_4036_MOESM2_ESM.docx]

PLAIN LANGUAGE STATEMENT AND CONSENT FORM

TO: Participants

Plain Language Statement

Date: June 2019

Full Project Title: Enhancing Treatment for Depression: A Randomised Trial of Adjunct Memory Specificity Training

Principal Researcher: Dr David Hallford

Associate Researcher(s): Anna Carmichael, Associate Professor David Austin, Dr Keisuke Takano, Professor Filip Raes, Associate Professor Matthew Fuller-Tyszkiewicz

1. Introduction

You are invited to take part in this research project. You have been invited as you have indicated you are experiencing symptoms of depression that might be higher than average in Australia. Research has shown that higher levels of depressive symptoms are related to more difficulty remembering different events from the past. Research also shows that improving the ability to recall memories can help to reduce depressive symptoms. This research project will assess if adding an online training program to improve the recall of personal memories, call Memory Specificity Training, will help to improve the support that you will already receive from the service you have gone to for help.

Please read this information carefully. Ask questions about anything that you don’t understand or want to know more about. Before deciding whether or not to take part, you might want to talk about it with a relative, or friend.

Participation in this research is voluntary. If you don’t wish to take part, you don’t have to.

If you decide you want to take part in the research project, then by clicking the “I agree” button below you are telling us that you:

•understand what you have read;

•consent to take part in the research project;

•consent to be involved in the procedures described;

•consent to the use of your personal information as described

2. What is the purpose of the research project?

The purpose of our project is to examine whether an online memory training program will improve the recall of personal memories, and reduce depressive symptoms. In particular, we are assessing whether this intervention leads to better outcomes when combined with the usual care you receive from a service. So, in our study everyone receives the normal support from the service they have sought help from (e.g., psychological therapy/counselling), but half of the participants will also have access to the online training program. We will then compare these groups to see if those who used the memory training program have better outcomes than those that did not.

3. What does participation in the research project involve?

If you participate in the study you will still receive usual care from the service you have sought support from. This will not be affected by taking part in this study. If you take part, then you will be randomly chosen to either receive your usual support plus have access to the memory training program immediately, or receive your usual support and have access to the program in 6 months. So, all participants will have access to the program, half now, and half later. The researchers will not have any influence on who is randomised to these groups.

All participants will first complete some questionnaires that are accessible online, these relate to your memory and your psychological well-being. Following this, some participants will have immediately have access to the memory specificity training program for a period of one months, and the other group in 6 months’ time. At the end of the one month period, all participants will be again asked to complete a series of questionnaires, and then again 3 and 6 months later. After completing the 6-month questionnaire, participants who did not already have access to the training program will then be given access. The questionnaires are expected to take approximately 30-45 minutes to complete.

The training program consists of 7 modules which are to be completed during this two week period. The modules consist of some brief education about memory recall and depression, and examples of how to recall detailed autobiographical memories. In each module you will be presented with a series of cue words to which you will be asked to recall personal memories. After responding to each cue word you will receive feedback that is designed to help you learn to retrieve memories in a more detailed way. The time taken to complete the training modules ranges from 10 to 30 minutes each. Participants in the intervention group will receive prompts to complete the training modules.

We encourage you to contact the researchers to answer any questions you might have. Participation in this study is purely online, and will not involve face-to-face support from the researchers. Taking part will not affect the treatment you receive from any other service.

4. What are the possible benefits?

Findings from this study will help us understand is adding this memory training program to usual care leads to improved outcomes. It is possible that participants will experience benefits for their mental health. Aside from this, no other benefits are anticipated for participants themselves.

5. What are the possible risks?

There is no risk of harm or discomfort anticipated. Should participants find themselves experiencing any distress, they are free to cease participation at any point.

6. Do I have to take part in this research project?

Participation in any research project is voluntary. If you do not wish to take part you are not obliged to. You are free to withdraw at any time during your involvement in the study. However, please note that when you submit the final questionnaire at the six-month follow-up, or the study is completed, the information you have provided will be linked using a code and de-identified. At this point, withdrawal of your information from the study will not be possible.

Your decision whether to take part or not to take part, or to take part and then withdraw, will not affect your relationship with Deakin University, or any of the services that you are seeking support from. These services will not be provided with information relating to your own, personal participation in the study.

Before you make your decision, a member of the research team will be available to answer any questions you have about the research project. You can ask for any information you want.  Click on the “I Agree” button below only after you have had a chance to ask your questions and have received satisfactory answers.

7. How will I be informed of the final results of this research project?

Once the project has been completed, a summary of findings will be available. The researchers listed at the bottom of this plain language statement may be contacted to obtain the results of this project, or participants may provide the researchers with a contact email and results can be sent to them when finalised. Final results are expected to be available by the middle of 2021.

8. What will happen to information about me?

Your information will remain confidential and will only be used for the purpose of this current research project. We plan to publish the results in a scientific journal. In any publication, information will be provided in such a way that you cannot be identified.

Any data you supply will be stored on password-protected computers at Deakin University for a minimum of five years from the date of research publication. The data will then be deleted. Only the research team will have access to the information

The data may also be uploaded to a publisher or an open access archive. In either of these circumstances, the data will be publicly available. However, it will be de-identified with only restricted demographic information about you made available. Only coded responses to the questionnaires will be uploaded (i.e., numbers), and not any personal, identifiable information. It would be most unlikely that you could be identified from your responses.

9. Can I access information about me?

In accordance with relevant Australian and/or Victorian privacy and other relevant laws, you have the right to access the information collected and stored by the researchers about you. However, please note that once the information you have provided is de-identified it will no longer be possible to access this information. Information collected in this project will be kept for at least five years from the date of publication, which is expected to occur in 2021

10. Is this research project approved?

The ethical aspects of this research project have been approved by the Deakin University Human Ethics Advisory Group in accordance with the principles of the National Statement on Ethical Conduct in Human Research (2007) produced by the National Health and Medical Research Council of Australia. This statement has been developed to protect the interests of people who agree to participate in human research studies. The researchers will also maintain a monitoring process of the study, involving ongoing consultation to identify and manage any potential ethical issues that may arise.

11. Who can I contact?

The person you may need to contact will depend on the nature of your query. Therefore, please note the following:

For further information:

If you want any further information concerning this project or if you have any problems which may be related to your involvement in the project (for example, feelings of distress), you can contact the principal researcher Dr David Hallford 9244 3742 or any of the following people:

Name:  Associate Professor David Austin

Role: Deakin University – Researcher

Telephone: 9244 3042

You may contact the following community services which can provide professional assistance:

Lifeline: 13 11 14. A 24-hour telephone crisis service available all day, every day of the year

Reachout.com: An Australian website providing practical information, tools, and support for young Australians

Complaints

If you have any complaints about any aspect of the project, the way it is being conducted or any questions about your rights as a research participant, then you may contact:

The Manager, Deakin Research Integrity, Deakin University, 221 Burwood Highway, Burwood Victoria 3125, Telephone: 9251 7129, research-ethics@deakin.edu.au

Please quote project number [2019_051].

Clicking the red button indicates that you have read and understood the information above and provide consent to participate in this study.
